# The effects of gastrodin injection on hypertension: A systematic review and meta-analysis

**DOI:** 10.1097/MD.0000000000020936

**Published:** 2020-07-02

**Authors:** Lichao Qian, Shihai Yan, Yizhuo Li, Lihua Wu, Yawei Zheng, Yixuan Wang, Zhuyuan Fang

**Affiliations:** a Affiliated Hospital of Nanjing University of Chinese Medicine; b Nanjing University of Chinese Medicine, Nanjing, Jiangsu, China.

**Keywords:** gastrodin injection, hypertension, meta-analysis, systematic review

## Abstract

**Background:**

Hypertension is a common chronic disease and poses a huge burden to health care systems. Recent studies have shown that gastrodin injection (GI) has a potential supplementary therapeutic effect on hypertension.

**Objectives:**

To systematically assess the efficacy and safety of GI in treatment of hypertension.

**Methods:**

Systematic search was conducted on 7 databases (PubMed, Cochrane Library, Embase, Wanfang database, China biomedical literature service system, VIP Chinese Sci-tech journal database and China national knowledge internet). The retrieval time was from the establishment of database to February 15, 2020. Two researchers independently selected literature, extracted data and evaluated the risk of bias in the study. The methodological quality was evaluated with Cochrane handbook. The meta-analysis was performed with Stata 14.0 software.

**Results:**

Thirteen studies were included in this study involving 1525 patients. Compared with using conventional therapy alone, GI combined with conventional therapy can decrease systolic blood pressure (weighted mean difference [WMD] −6.67, 95% confidence interval [CI]: −10.30, −3.04. number of estimates [k] = 9, *I*^2^ = 89.3%), diastolic blood pressure (WMD −4.52, 95% CI: −7.79, −1.26. k = 9, *I*^2^ = 92.3%), and improve the clinical efficacy (relative risk [RR] 1.18, 95% CI: 1.10, 1.26. k = 6, *I*^2^ = 12.6%).

**Conclusions:**

The current evidence showed that GI combined with conventional therapy can improve systolic blood pressure, diastolic blood pressure and clinical efficacy. GI can become a supplementary treatment for hypertension.

## Abstract 2

Supplemental Digital Content is available in the text

## 1 Introduction

Hypertension is one of the most common cardiovascular diseases and has become a huge threat to human health.^[1]^ According to the 2012 World Health Statistics report, one-third of the worlds adults were suffering from hypertension, contributing to about half of the total deaths caused by stroke and heart diseases.^[2]^ The pathobiology of hypertension is complex, including imbalance of renin-angiotensin-aldosterone system (RAAS), excessive sodium, dysfunction of vascular endothelial function, increase of blood viscosity, and many other mechanisms.^[3]^ Among them, angiotension II (Ang II) has a strong contractive effect on blood vessels and is a main factor for the maintenance and deterioration of hypertension.^[4]^ Endothelin (ET) can increase the flow of Ca^2+^, thus enhancing the pressor effect of other vasoconstrictors and reducing the synthesis of nitric oxide (NO), and destroy the antagonistic effect of both on maintaining the basic tension of blood vessels, which promotes the development of hypertension.^[5]^ Several kinds of antihypertension medications have been proved to have therapeutic effects of lowering the blood pressure (BP). However, some patients still had serious side effects and did not have a good response to the existing conventional therapy. There is a demand to find an alternative therapy that may benefit these patients.

In recent years, traditional Chinese medicine (TCM) has shown a unique role in the treatment of hypertension.^[6–9]^ According to the theory of TCM, hypertension usually belongs to vertigo and headache.^[6,10]^ Gastrodin is the extract of Gastrodia elata, a famous TCM, which has the functions of stopping spasm caused by wind, calming the liver-yang, removing wind, and dredging collaterals and it is mainly used to treat dizziness and headache.^[11]^ Modern pharmacological researches show that GI can improve the function of vascular endothelial cells, inhibit the stimulation of sympathetic nervous system and reduce the level of Ang II, aldosterone (ALD), and angiotensin type 1 receptor (AT1R) so as to intervene the RAAS to reduce BP.^[12–15]^ It can also antagonize the vasoconstriction effect of catecholamine transmitters, non-competitively antagonize the potential dependent calcium channels and receptor-operated calcium channels and prevent the influx and release of Ca^2+^, leading to vasodilation and lowering BP.^[14]^ In the past decades, numerous clinical studies using GI as a supplementary treatment to cure hypertension reported good effects.^[12,16–27]^ However, there were differences in the results of related clinical trials. We collected all relevant available studies to carry out the meta-analysis. The main purpose of this study is to assess the efficacy and safety of GI combined with conventional therapy on hypertension.

## 2 Materials and methods

### 2.1 Search strategy and study eligibility

Our systematic review and meta-analysis was undertaken according to the PRISMA guidelines.^[28]^ All analyses were based on previous published studies, thus no ethical approval and patient consent were required. We searched the following databases for relevant studies from the establishment date of the database to February 15, 2020: PubMed, Cochrane Library, Embase, Wanfang database, China biomedical literature service system, VIP Chinese Sci-tech journal database and China national knowledge internet. And we collected randomized controlled trials (RCTs) of GI combined with conventional therapy for hypertension. The search terms employed were as follows: (“gastrodin’” OR “gastrodine” OR “Tianxuanqing”) AND (“Hypertension, Essential” OR “Essential Hypertension” OR “Primary Hypertension” OR “Hypertension” OR “EH” OR “Blood pressure”), along with a combination of MeSH indexing approaches. There was no language limitation. At the same time, we also screened the references of the included articles to ensure that we can retrieve the relevant articles more comprehensively.

### 2.2 Inclusion/exclusion criteria

Studies that met the following criteria were included:

1. RCTs on hypertensive patients;
2. Conventional treatment as intervention measures in control group, GI plus conventional treatment as intervention measures in combination group;
3. At least 1 of the relative outcomes or adverse effect was reported.

Systolic blood pressure (SBP), diastolic blood pressure (DBP), and clinical efficacy were primary outcomes. ET, NO, and pulse pressure difference (PP) were secondary outcomes. When multiple publications reporting data from the same trial were available, only the study with the largest sample size was included. Single case reports, animal studies and studies of basic science were not included. Language was not restricted in this meta-analysis to minimize the publication bias.

### 2.3 Data extraction and bias/quality assessment

Two investigators reviewed the literature, extracted data and evaluated them independently. When there were disagreements, the decision was made by discussion or consultation to a third investigator. Information including author, year of publication, patients characteristics, intervention measures, intervention period, outcomes (DBP, SBP, clinical efficacy, ET, NO, PP) and adverse reactions were retreived from each study. The corresponding author of the original study was contacted by email when the data was incomplete or there was any uncertainty in the publications. We used the “risk of bias” evaluation tool in Cochrane Handbook to evaluate the quality of included studies, including the following aspects: random sequence generation, allocation concealment, blinding of participants and personnel, blinding of outcome assessment, incomplete outcome date, selective reporting, and bias from other sources. Each of these aspects was evaluated separately and the following categories were used by us: low risk, high risk, and unclear.

### 2.4 Analysis

Relative risk (RR) and its 95% confidence interval (CI) were used to represent the curative effect analysis statistics for count data, and the weighted mean difference (WMD) and its 95% CI were used as the analysis statistic to represent continuous changes. We extracted RRs, WMDs and those 95% CIs from the publications, where available. When necessary, we calculated WMDs/CIs from original study data provided in the publication. We used *I*^2^ statistics and *P* value to evaluate the level of heterogeneity between the included studies. When *I*^2^ > 50% or *P* < .05, we chose the random effect model. Otherwise, the fixed effect model was chosen. Subgroup analyses were conducted to explore the potential sources of heterogeneity. Sensitivity analyses were conducted by removing each estimate one at a time and recalculating the pooled estimates to detect potential outliers. Funnel plots and Egger tests were used to determine whether there was publication bias. Meta-analysis was undertaken with Stata 14.0 software.^[29]^

## 3 Results

### 3.1 Search results and study characteristics

**Figure 1:**
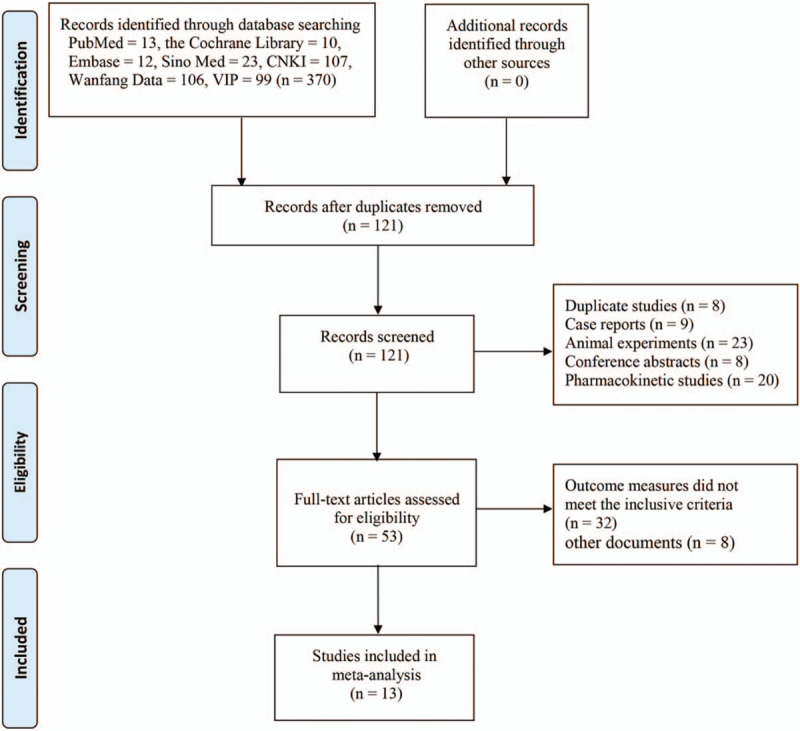
Flow-process diagram of literature retrieval.

**Figure 2:**
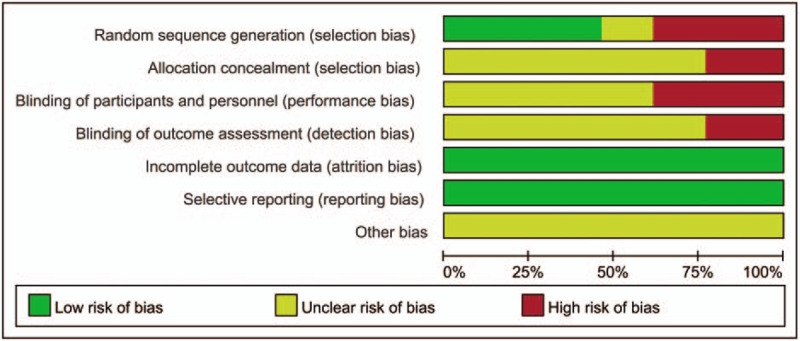
Risk of bias.

**Figure 3:**
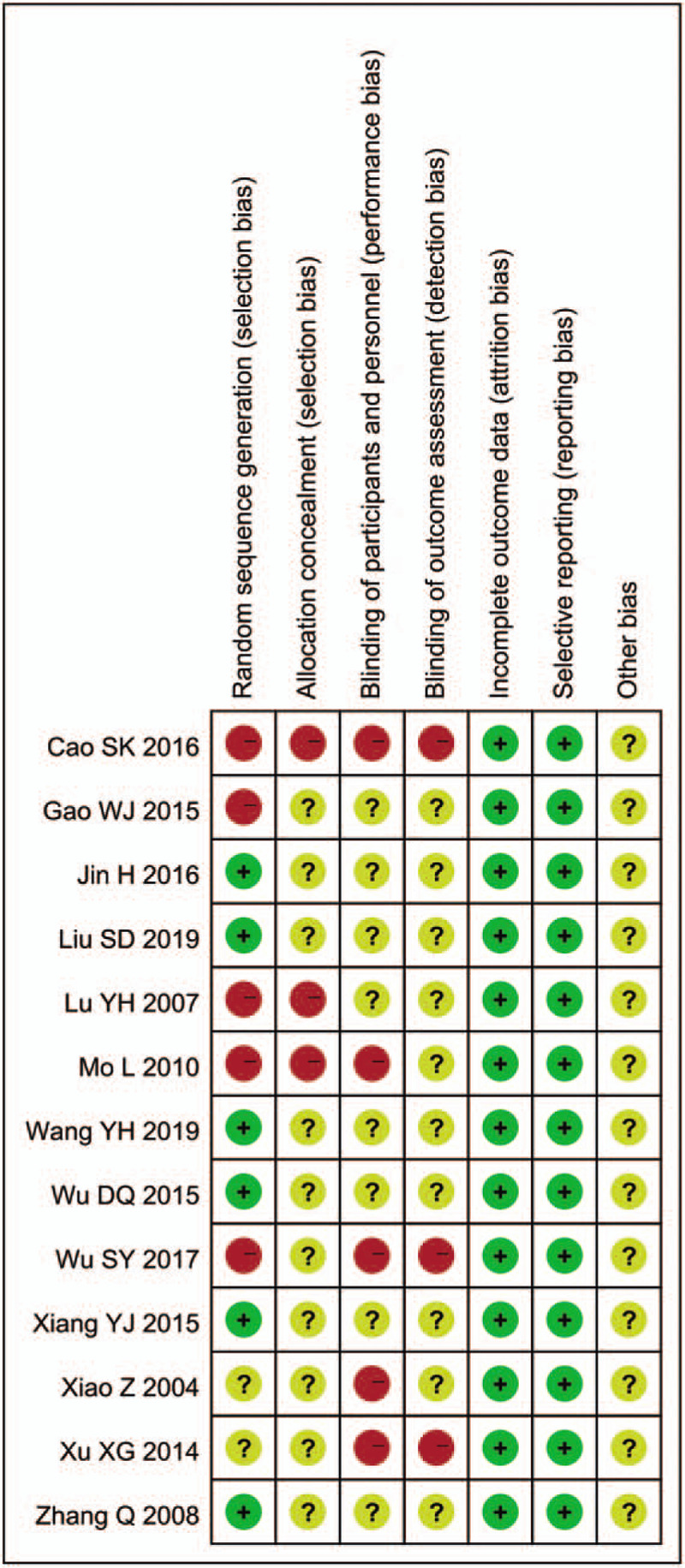
Risk of bias summary and graph.

A total of 370 related literatures meeting the inclusion criteria were enrolled for the initial examination. 249 duplications were excluded. Then 121 related literatures were carefully reviewed by reading their titles and abstracts. Sixty eight articles further were excluded because of the following reasons: duplicate studies (n = 8), case reports (n = 9), animal experiments (n = 23), conference abstracts (n = 8), and pharmacokinetic studies (n = 20). The full text of the remaining 53 articles were reviewed and 40 of them were further excluded because of the following reasons: outcome measures did not meet the inclusion criteria (n = 32), other documents (n = 8). A total of 13 RCTs^[12,16–27]^ containing 1525 patients were included in the final meta-analysis. The process and results of literature screening are shown in Figure 1. There are 766 patients in the combination group while 759 patients in control group. All of these trials were carried out in the hospitals of China. Included studies were published between 2004 and 2019. The sample sizes ranged from 63 to 220. Durations of the interventions ranged from 2 weeks to 3 months. The conventional therapy of the included studies included amlodipine, valsartan, benazepril, levamlodipine besylate, nifedipine, etc. The basic characteristics of the included studies are shown in Table 1. The quality of the included studies was relatively low. The assessment of bias risk and quality is shown in Figures 2 and 3.

**Table 1:**
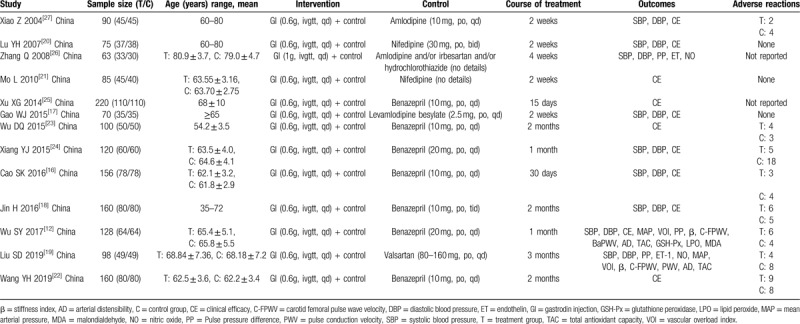
Characteristics of 13 studies fulfilling the inclusion criteria.

### 3.2 Primary outcome measures

#### 3.2.1 Systolic blood pressure (SBP, mm Hg)

**Figure 4:**
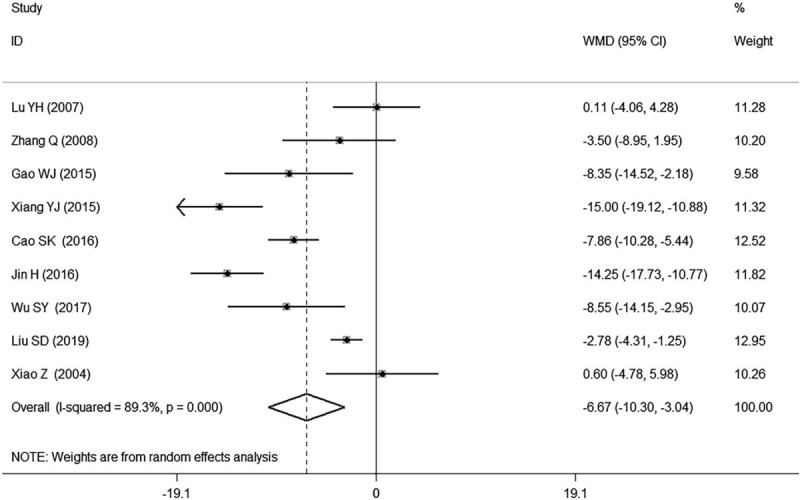
Forest plot of SBP of GI combined with conventional therapy vs conventional therapy.

Nine studies^[12,16–20,24,26,27]^ involving 992 participants reported the results of SBP in patients with hypertension. Six of them^[12,16–19,24]^ found GI plus conventional treatment had a significant decrease of SBP in patients with hypertension while the remaining 3 of them^[20,26,27]^ reported non-significant association. The meta-analysis indicated that GI combined with conventional therapy had a significant reduction of SBP compared with using conventional therapy alone in patients with hypertension (WMD −6.67, 95% CI: −10.30, −3.04. number of estimates [k] = 9, *I*^2^ = 89.3%, Figure 4).

#### 3.2.2 Diastolic blood pressure (DBP, mm Hg)

**Figure 5:**
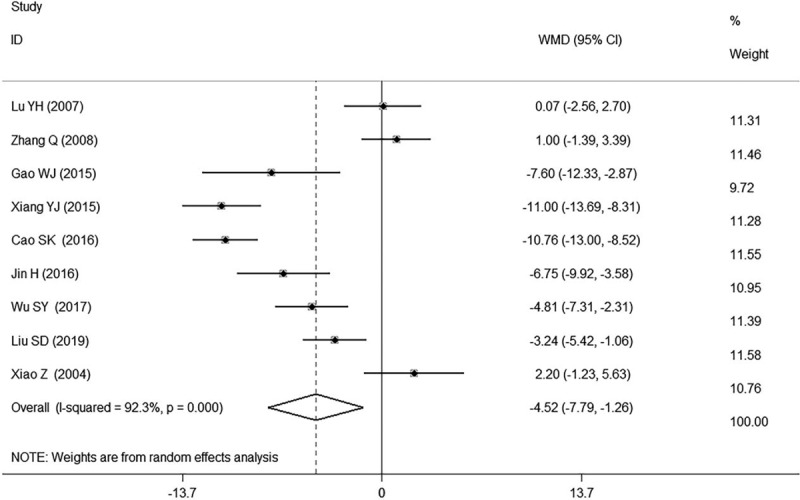
Forest plot of DBP of GI combined with conventional therapy vs conventional therapy.

Nine studies^[12,16–20,24,26,27]^ reported the results of the DBP. Six of them^[12,16–19,24]^ found GI plus conventional treatment had a significant decrease of DBP in patients with hypertension while the remaining 3 of them^[20,26,27]^ reported non-significant association. The meta-analysis indicated that GI combined with conventional therapy had a significant reduction of DBP compared with using conventional therapy alone in patients with hypertension (WMD −4.52, 95% CI: −7.79, −1.26. k = 9, *I*^2^ = 92.3%, Fig. 5).

### 3.3 Clinical efficacy

**Figure 6:**
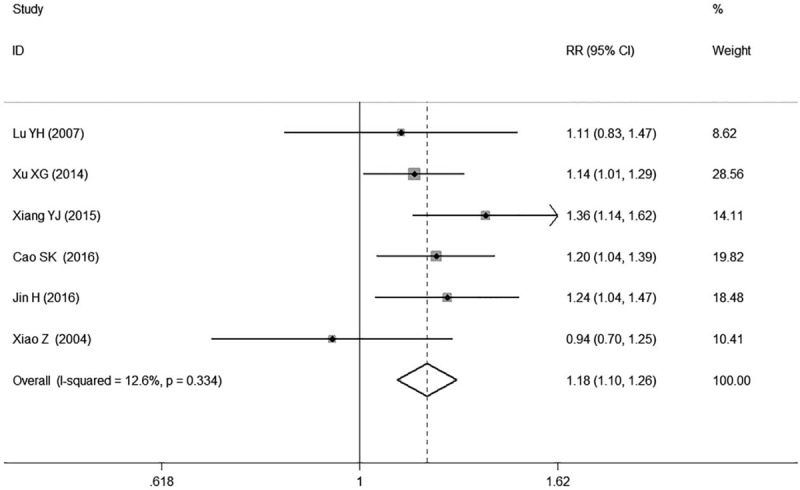
Forest plot of clinical efficacy of GI combined with conventional therapy vs conventional therapy.

A total of 6 studies^[16,18,20,24,25,27]^ reported the results of the total effective rate of lowing BP, involving 821 patients (410 in the combination group and 411 in the control group). Four of them^[16,18,24,25]^ found GI plus conventional treatment had a significant increase of clinical efficacy in patients with hypertension while the remaining 2 of them^[20,27]^ reported non-significant association. Our meta-analysis indicated that GI combined with conventional therapy had a significant increase of clinical efficacy compared with using conventional medicine alone in patients with hypertension (RR 1.18, 95% CI: 1.10, 1.26. k = 6, *I*^2^ = 12.6%, Fig. 6).

### 3.4 Subgroup analysis

In subgroup analysis for different durations of treatment, the results showed a non-significant difference in SBP, DBP and clinical efficacy between the combination treatment group and the control group at the 2-week follow-up time point (SBP: WMD −2.21, 95% CI: −7.36, 2.93. k = 3, *I*^2^ = 66.0%; DBP: WMD −1.46, 95% CI: −6.26, 3.34. k = 3, *I*^2^ = 82.2%; clinical efficacy: RR 1.09, 95% CI: 0.98, 1.21. k = 3, *I*^2^ = 0.0%, Table 2, Supplemental Digital Content Figs. S1, S3 and S5). However the combination treatment showed a significant reduction in SBP, DBP, and clinical efficacy when the observation time was more than 4 weeks (SBP: WMD −8.63, 95% CI: −13.13, −4.14. k = 6, *I*^2^ = 91.8%; DBP: WMD −5.91, 95% CI: −9.72, −2.09. k = 6, *I*^2^ = 92.9%; clinical efficacy: RR 1.26, 95% CI: 1.14, 1.38. k = 3, *I*^2^ = 0.0%, Table 2, Supplemental Digital Content Figs. S1, S3, and S5).

**Table 2:**
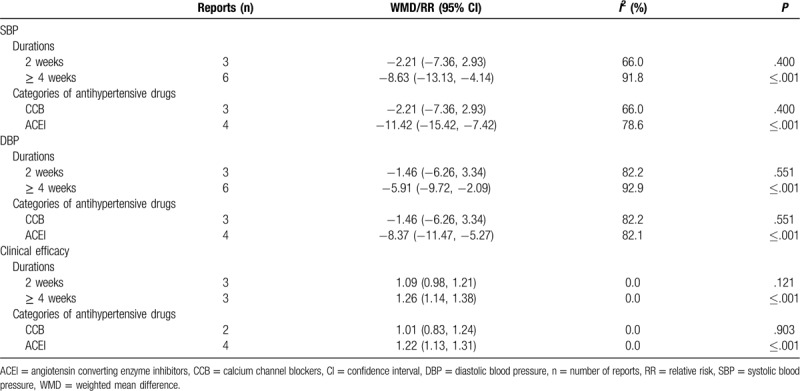
Subgroup analyses of SBP, DBP and clinical efficacy.

In subgroup analysis for different types of antihypertensive drugs, the results showed a non-significant difference in SBP, DBP, and clinical efficacy between the combination group and the control group with calcium channel blockers (CCB) (SBP: WMD −2.21, 95% CI: −7.36, 2.93. k = 3, *I*^2^ = 66.0%; DBP: WMD −1.46, 95% CI: −6.26, 3.34. k = 3, *I*^2^ = 82.2%; clinical efficacy: RR 1.01, 95% CI: 0.83, 1.24. k = 2, *I*^2^ = 0.0%, Table 2, Supplemental Digital Content Figs. S2, S4, and S6). However, the combination treatment showed a significant reduction in SBP, DBP and clinical efficacy with angiotensin converting enzyme inhibitors (ACEI) (SBP: WMD −11.42, 95% CI: −15.42, −7.42. k = 4, *I*^2^ = 78.6%; DBP: WMD −8.37, 95% CI: −11.47, −5.27. k = 4, *I*^2^ = 82.1%; clinical efficacy: RR 1.22, 95% CI: 1.13, 1.31. k = 4, *I*^2^ = 0.0%, Table 2, Supplemental Digital Content Figs. S2, S4, and S6).

### 3.5 Secondary outcome measures

Three of studies^[12,19,26]^ found GI plus conventional treatment had a significant decrease of PP in patients with hypertension (WMD −6.00, 95% CI: −8.07, −3.93. k = 3, *I*^2^ = 60.0%, Table 3 and Supplemental Digital Content Fig. S7). Two of studies^[19,26]^ found GI plus conventional treatment had a significant decrease of ET (WMD −8.64, 95% CI: −13.09, −4.19, k = 2, *I*^2^ = 0.0%, Table 3 and Supplemental Digital Content Fig. S8) and a significant increase of NO in patients with hypertension (WMD 6.86, 95% CI: 5.45, 8.27. k = 2, *I*^2^ = 0.0%, Table 3 and Supplemental Digital Content Fig. S9).

**Table 3:**
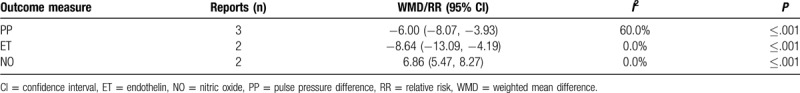
Meta-analyses of secondary outcome measures.

### 3.6 Safety

Eleven of the included studies reported adverse events.^[12,16–24,27]^ There were 8 cases of digestive tract reaction, 8 cases of nausea and vomiting, 8 cases of cough, 6 cases of flushing, 5 cases of rash, 4 cases of nausea and vomiting, 4 cases of vertigo, 4 cases of asthenia, 3 cases of cough, 2 cases of mild edema of ankle, 2 cases of lower extremity edema, and 1 case of headache in combination therapy groups. There were 13 cases of vertigo, 11 cases of flushing, 6 cases of digestive tract reaction, 4 cases of asthenia, 2 cases of flushing, and 2 cases of mild ankle edema in control groups. Three studies^[17,20,21]^ reported that there were no adverse events related to combination therapy. The remaining 2^[25,26]^ did not report adverse events in their studies. All these symptoms were relieved after symptomatic treatment and did not affect the implementation of the trials.

### 3.7 Sensitivity analysis and publication bias

We found high heterogeneity according to the test for heterogeneity of SBP and DBP (SBP: *I*^*2*^ = 89.3%, DBP: *I*^*2*^ = 92.3%, Figs. 4 and 5), and the random effect model was used. The heterogeneity in the results of clinical efficacy was low (*I*^*2*^ = 12.6%, Fig. 6), and fixed effect model was used. We used the sensitivity analyses to judge the stability of the results and 1 study^[16]^ for DBP had some effect on the pooled association estimate (Supplemental Digital Content Figs. S10, S11, and S12). Funnel plots and Egger tests were used to determine the publication bias, and no publication bias was found (Egger tests: SBP: *P* = 0.420, DBP: *P* = 0.916, clinical efficacy: *P* = 0.500, Supplemental Digital Content Table S1, Figs. S13, S14, and S15).

## 4 Discussion

This meta-analysis found that the level of SBP, DBP, PP, and ET in patients treated with combination therapy were significantly lower than those in patients treated with conventional therapy alone, and the clinical efficacy and level of NO was significantly higher in patients treated with combination therapy when compared with patients in the control group. This meta-analysis suggested that GI can be an effective supplementary treatment for hypertension.

Different antihypertensive drugs decrease the BP through different mechanisms (eg, reducing blood volume, inhibiting blood flow, inhibiting RAAS, blocking Ca^2+^ flow, reducing peripheral resistance, etc.).^[30]^ Although modern medications have certain curative effects in the treatment of hypertension, the symptoms in some patients still cannot be relieved to a satisfying level. Our finding indicated GI combined with conventional medicine had positive effects on lowering the BP in patients with hypertension. Gastrodin is the extract of Gastrodia elata. It has the effect of calming wind, reducing BP, clearing heat, activating blood circulation, and calming nerves in the theory of TCM.^[11]^ GI can regulate the BP through inhibiting RAAS system and preventing the influx and release of Ca^2+^.^[14,15]^ It indicated the combination of GI with conventional therapy may have a synergistic effect through above mechanisms.

In subgroup analyses for different intervention durations, we found that the treatment course longer than 4 weeks can lead to a significant reduction of BP. Hypertension is a chronic disease with complex etiologies and it usually takes a certain course before the treatment come into effect. The synergistic effect of GI combined with conventional treatment may accumulate with time. In subgroup meta-analysis for GI combined with different types of antihypertensive drugs, we found that patients using GI combined with ACEI had a significant reduction in BP. It has been reported^[12,14,15,31]^ that gastrodin can inhibit the stimulation of sympathetic nervous system, reduce the concentration of serum AngII, ALD, and AT1R so as to effectively inhibit the RAAS and peroxisome proliferator-activated receptor γ to reduce the BP. Chen HH reported^[32]^ gastrodin can decrease the levels of superoxide dismutase and malondialdehyde, protect the vascular through reducing lipid peroxidation injury and reduce BP in hypertensive rats. GI can also improve the hemodynamic state, protect the function of vascular endothelial cells, inhibit the synthesis of endothelin and dilate vessels. All of the above mentioned mechanisms work together to reduce BP.^[13,33,34]^ ACEI is one of the commonly used antihypertensive drugs. It can reduce BP by affecting RAAS. It can reduce the production of Ang II, inhibit sympathetic excitation, and regulate the function of vascular endothelium. The combination of GI and ACEI may produce a synergistic effect of lowering the BP through these mechanisms. The conventional medications reported in the included studies only cover CCB, ACEI, and ARB. The effects of GI combining with other categories of conventional medications (eg, beta blockers or diuretics or renin inhibitors) are unclear. Studies including more types of antihypertensive drugs with large samples are needed to supply our findings.

Our meta-analysis indicated GI plus conventional treatment had a significant decrease of ET and increase of NO in patients with hypertension. ET can increase the flow of Ca^2+^ and reduce the synthesis of NO to promote the occurrence and development of hypertension.^[4]^ Modern pharmaceutical studies have shown that GI can decrease the BP by protecting the function of vascular endothelial cells and inhibiting the synthesis of ET.^[13,33,34]^ The results of this study suggested that the antihypertensive effect of GI may be achieved by inducing vasodilatation and balancing the plasma levels of NO and ET and intervening the RAAS.^[33,35–37]^

There were some limitations in the meta-analysis. The sample size of the included studies was generally small. Medications used in the conventional therapy of the included studies only include CCB, ACEI, ARB and the effect of GI combined with other conventional therapy are unclear. More types of antihypertensive drugs are needed to make the finding more generally applicable. The quality of the included studies is relatively low, therefore careful cautions should be paid in interpretation of the results. The types of intervention methods and the length of intervention time were not uniform, which may affect the general applicability of the results.

## 5 Conclusions

Our meta-analysis indicated that GI combined with conventional therapy can reduce BP, improve endothelial function and clinical symptoms of patients with hypertension. It can improve the quality of life and extend the lifetime of patients with hypertension.

## Author contributions

**Conceptualization:** Lichao Qian, Zhuyuan Fang.

**Data curation:** Shihai Yan, Yizhuo Li.

**Formal analysis:** Lihua Wu, Yawei Zheng, Yixuan Wang.

**Project administration:** Lichao Qian, Zhuyuan Fang.

**Supervision:** Zhuyuan Fang, Shihai Yan.

**Writing – original draft:** Lichao Qian.

**Writing – review & editing:** Yizhuo Li, Shihai Yan.

## Supplementary Material

Supplemental Digital Content
