# Supplementary material for: The effects of gastrodin injection on hypertension: A systematic review and meta-analysis
Source: Medicine (Baltimore). 2020 Jul 2;99(27):e20936. doi: 10.1097/MD.0000000000020936 (PMC7337477; doi:10.1097/MD.0000000000020936)
Supplement: Supplemental Digital Content [file medi-99-e20936-s001.docx]

**Supplemental Digital Content**

[Supplemental Digital Content (Table S1): Egger’s test to evaluate publication bias. 2](#_Toc16373)

[Supplemental Digital Content (Figure S1): Subgroup analysis of SBP of GI combined with conventional therapy vs conventional therapy for treatment duration. 2](#_Toc25370)

[Supplemental Digital Content (Figure S2): Subgroup analysis of SBP of GI combined with conventional therapy vs conventional therapy for categories of antihypertensive agents. 3](#_Toc4394)

[Supplemental Digital Content (Figure S3): Subgroup analysis of DBP of GI combined with conventional therapy vs conventional therapy for treatment duration. 4](#_Toc6449)

[Supplemental Digital Content (Figure S4): Subgroup analysis of DBP of GI combined with conventional therapy vs conventional therapy for categories of antihypertensive agents. 5](#_Toc109)

[Supplemental Digital Content (Figure S5): Subgroup analysis of clinical efficacy of GI combined with conventional therapy vs conventional therapy for treatment duration. 6](#_Toc27379)

[Supplemental Digital Content (Figure S6): Subgroup analysis of clinical efficacy of GI combined with conventional therapy vs conventional therapy for categories of antihypertensive agents. 7](#_Toc12321)

[Supplemental Digital Content (Figure S7): Forest plot of PP of GI combined with conventional therapy vs conventional therapy. 8](#_Toc25094)

[Supplemental Digital Content (Figure S8): Forest plot of ET of GI combined with conventional therapy vs conventional therapy. 9](#_Toc22777)

[Supplemental Digital Content (Figure S9): Forest plot of NO of GI combined with conventional therapy vs conventional therapy. 10](#_Toc23403)

[Supplemental Digital Content (Figure S10): Sensitivity analysis for SBP. 11](#_Toc13988)

[Supplemental Digital Content (Figure S11): sensitivity analysis for DBP. 12](#_Toc29695)

[Supplemental Digital Content (Figure S12): Sensitivity analysis for clinical efficacy. 13](#_Toc10956)

[Supplemental Digital Content (Figure S13): Funnel plot for SBP. 14](#_Toc26108)

[Supplemental Digital Content (Figure S14): Funnel plot for DBP. 15](#_Toc20756)

[Supplemental Digital Content (Figure S15): Funnel plot for clinical efficacy. 16](#_Toc18308)

# Supplemental Digital Content (Table S1): Egger’s test to evaluate publication bias.

| **Outcome** | **Egger’s test (*P*)** |
| --- | --- |
| SBP | 0.420 |
| DBP | 0.916 |
| Clinical efficacy | 0.500 |


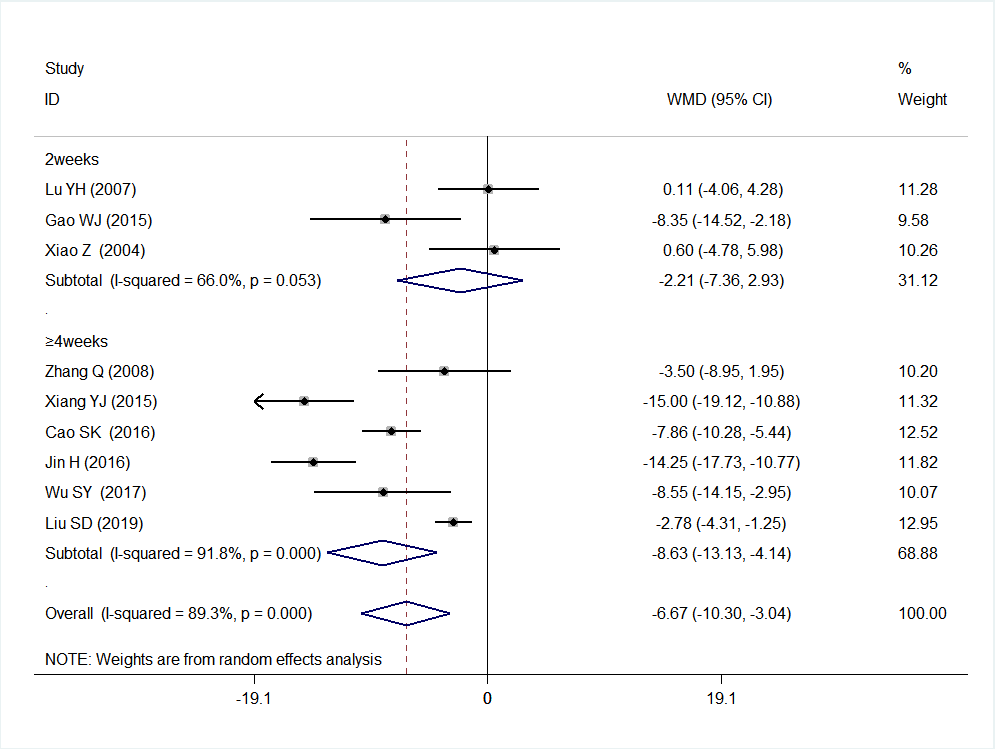


# Supplemental Digital Content (Figure S1): Subgroup analysis of SBP of GI combined with conventional therapy vs conventional therapy for treatment duration.


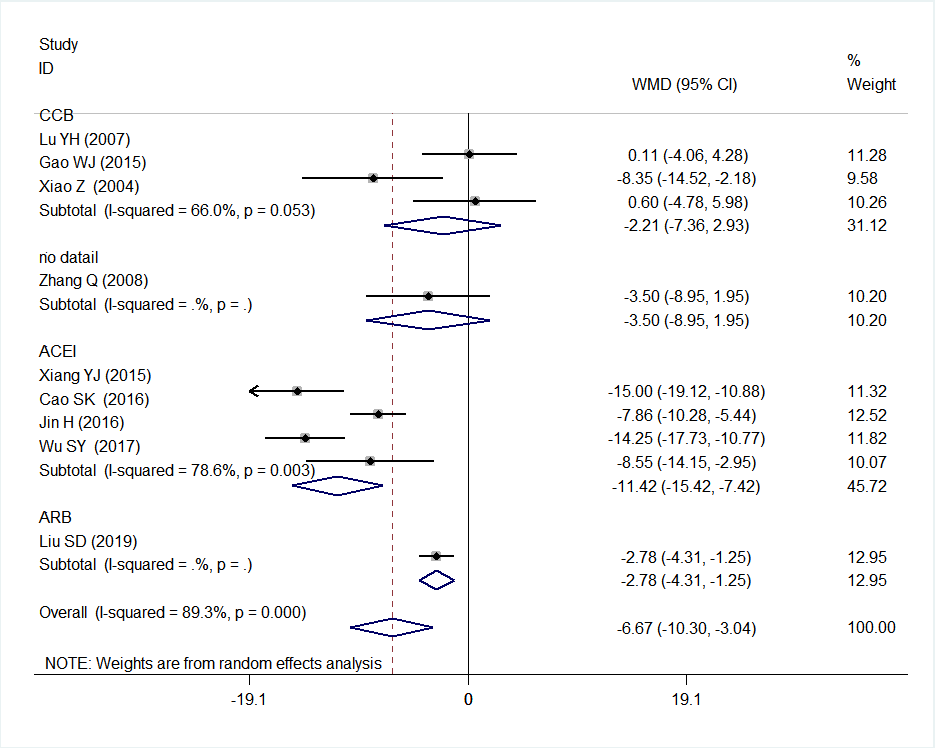


# Supplemental Digital Content (Figure S2): Subgroup analysis of SBP of GI combined with conventional therapy vs conventional therapy for categories of antihypertensive agents.


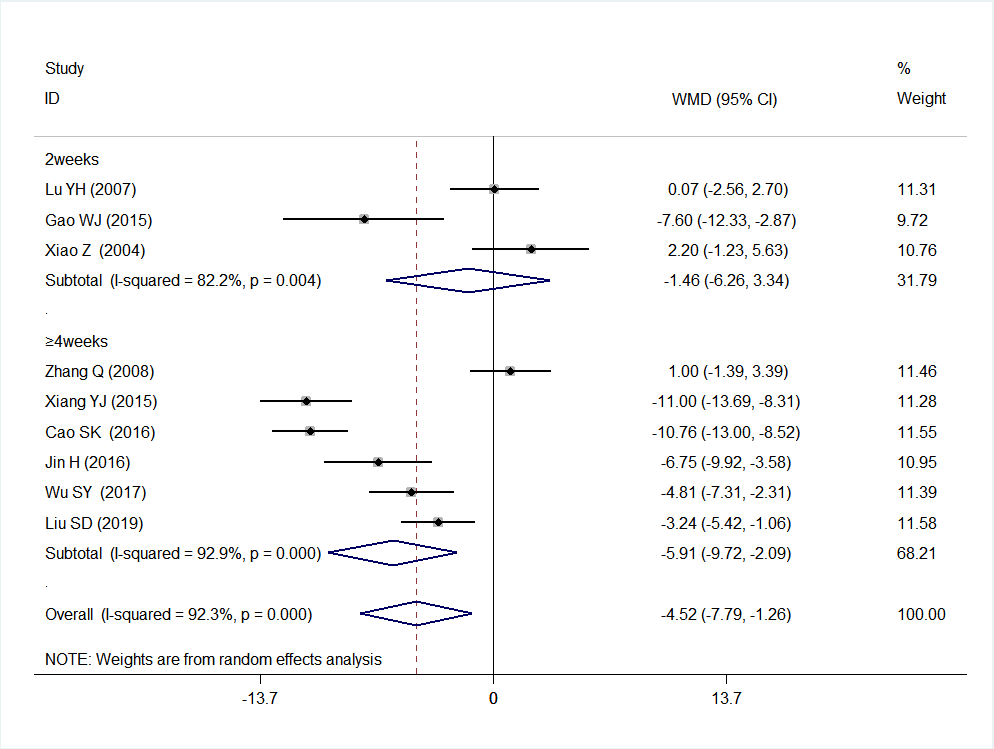


# Supplemental Digital Content (Figure S3): Subgroup analysis of DBP of GI combined with conventional therapy vs conventional therapy for treatment duration.

**
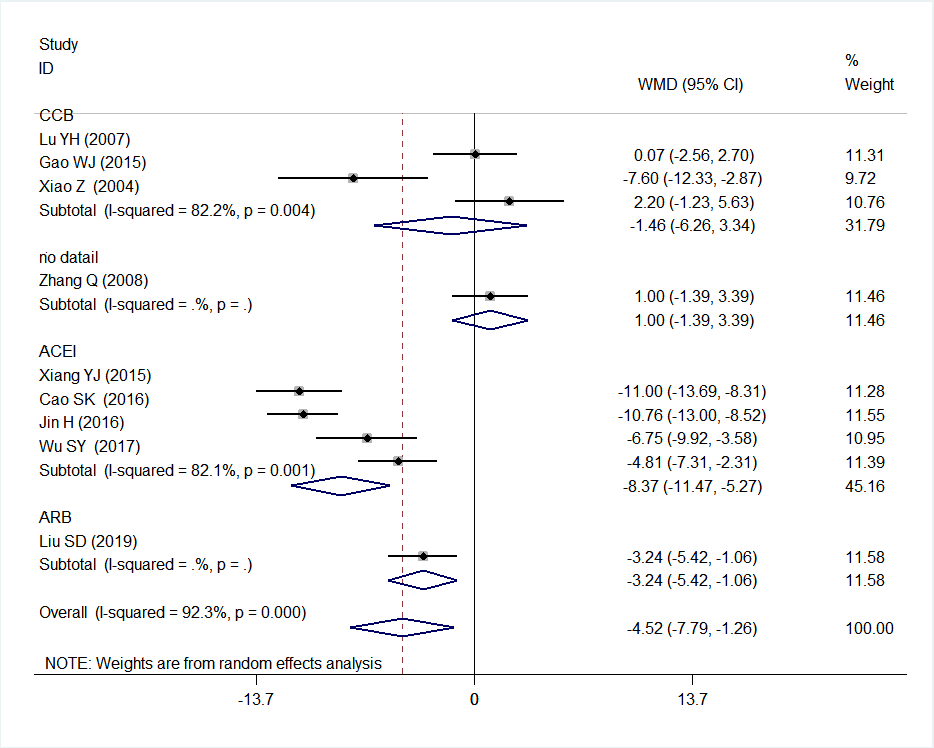
**

# Supplemental Digital Content (Figure S4): Subgroup analysis of DBP of GI combined with conventional therapy vs conventional therapy for categories of antihypertensive agents.

**
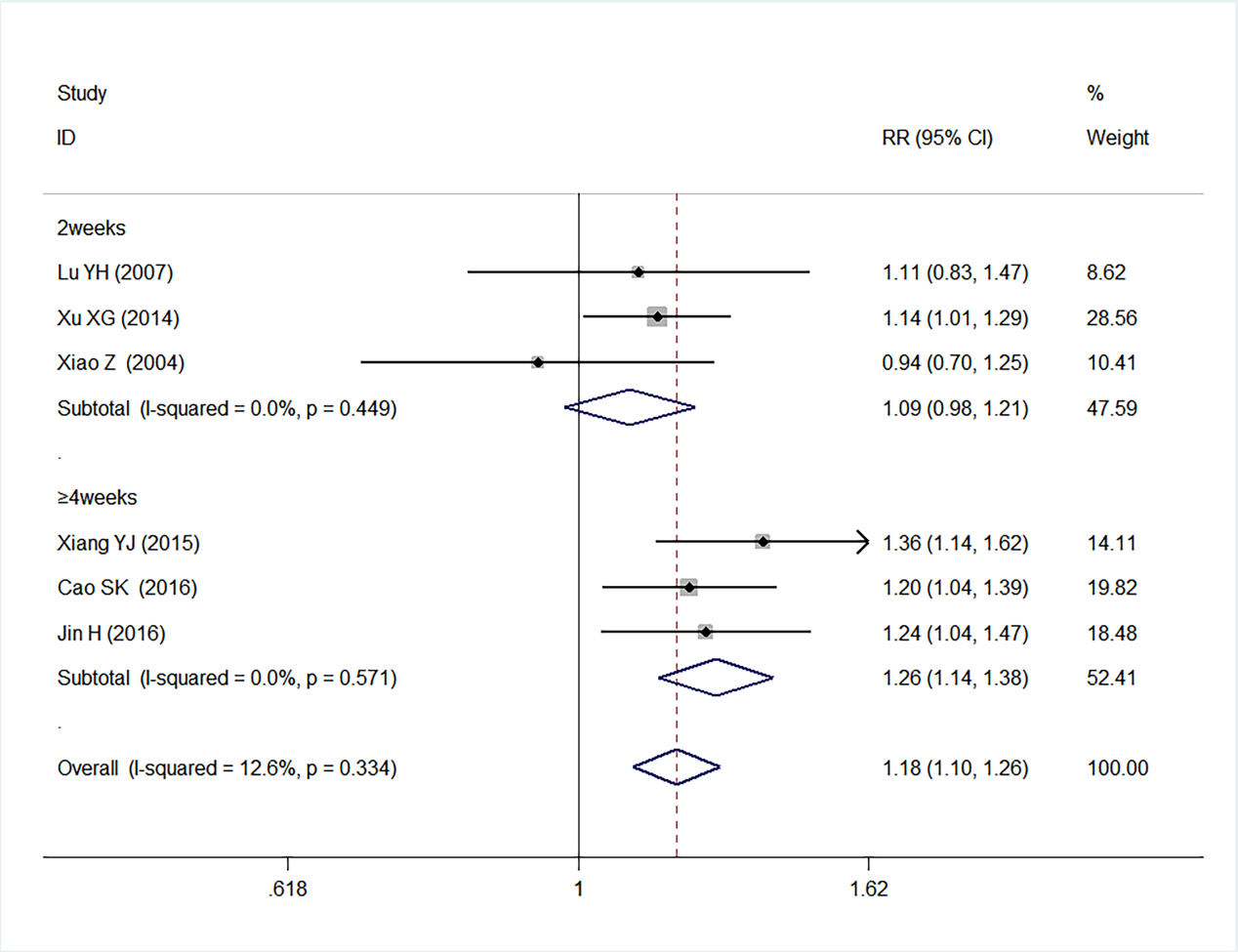
**

# Supplemental Digital Content (Figure S5): Subgroup analysis of clinical efficacy of GI combined with conventional therapy vs conventional therapy for treatment duration.

**
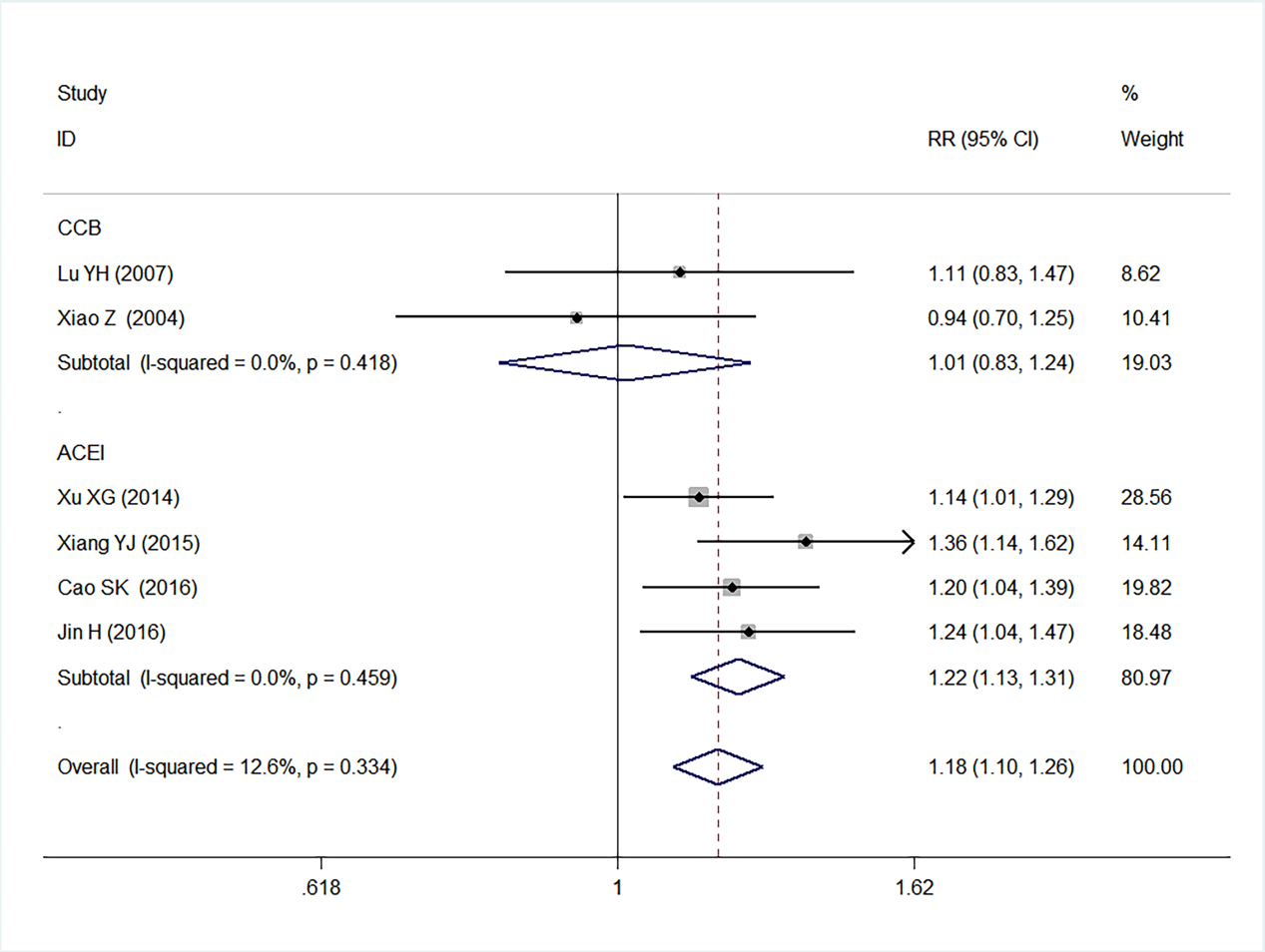
**

# Supplemental Digital Content (Figure S6): Subgroup analysis of clinical efficacy of GI combined with conventional therapy vs conventional therapy for categories of antihypertensive agents.

**
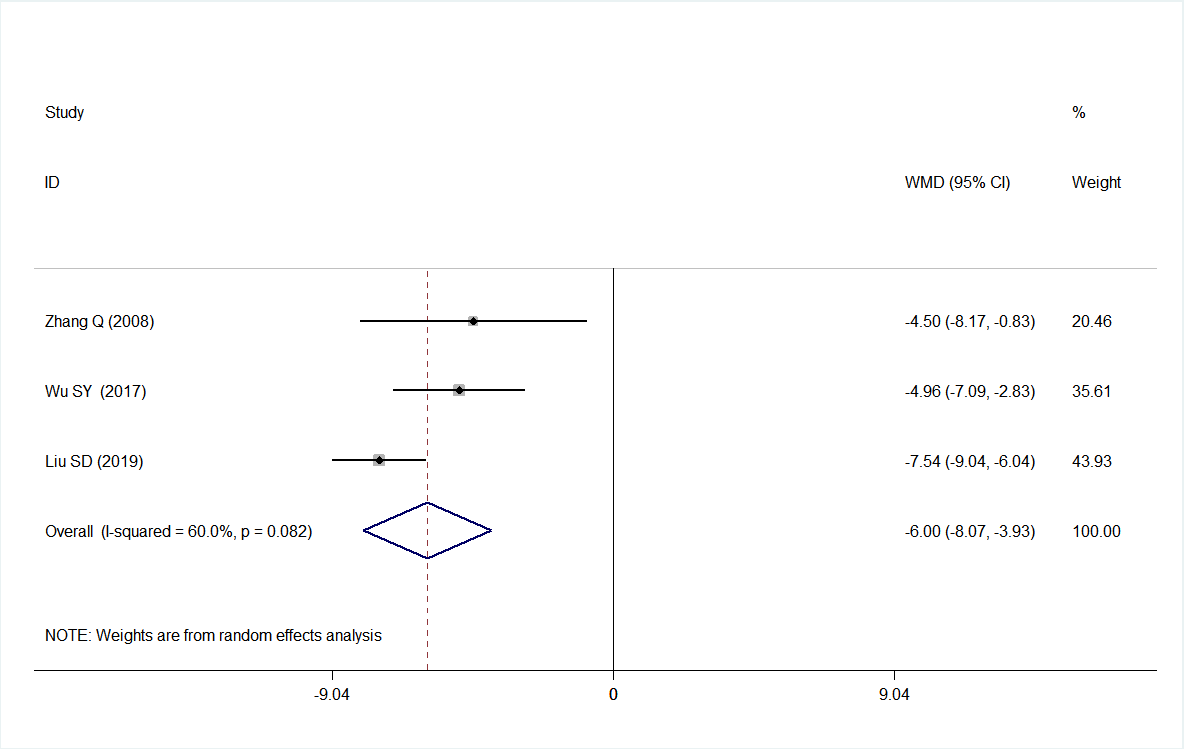
**

# Supplemental Digital Content (Figure S7): Forest plot of PP of GI combined with conventional therapy vs conventional therapy.

**
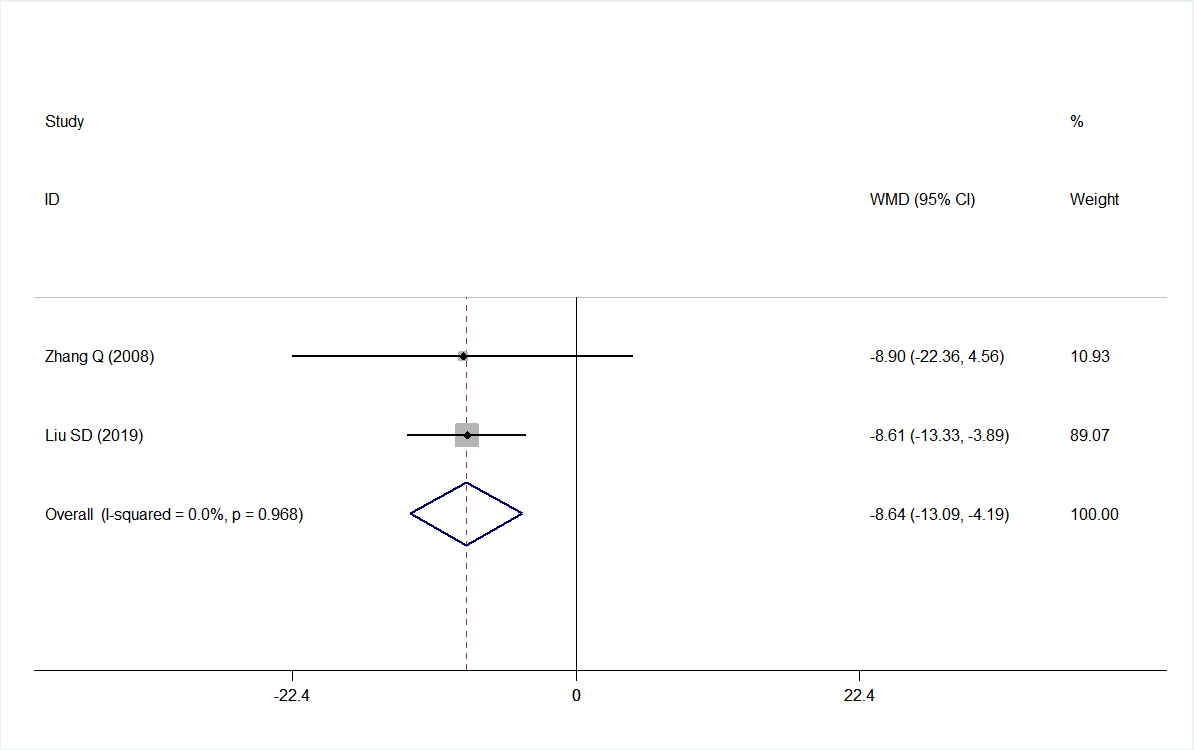
**

# Supplemental Digital Content (Figure S8): Forest plot of ET of GI combined with conventional therapy vs conventional therapy.

**
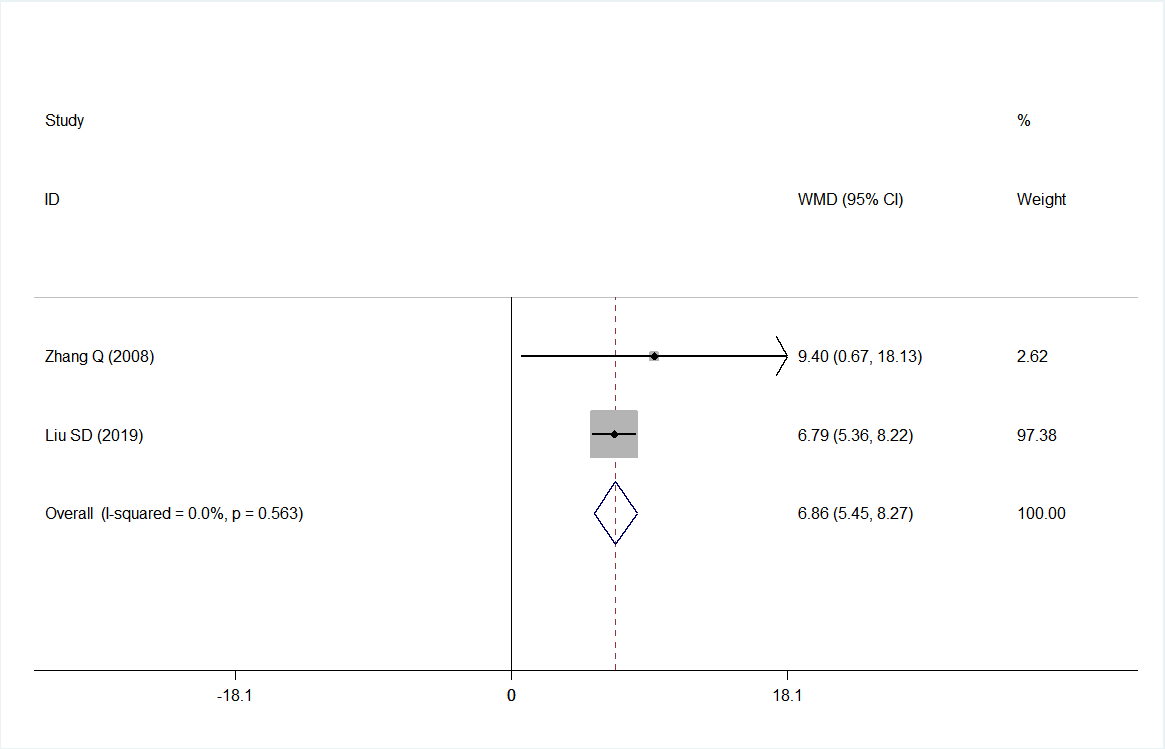
**

# Supplemental Digital Content (Figure S9): Forest plot of NO of GI combined with conventional therapy vs conventional therapy.

**
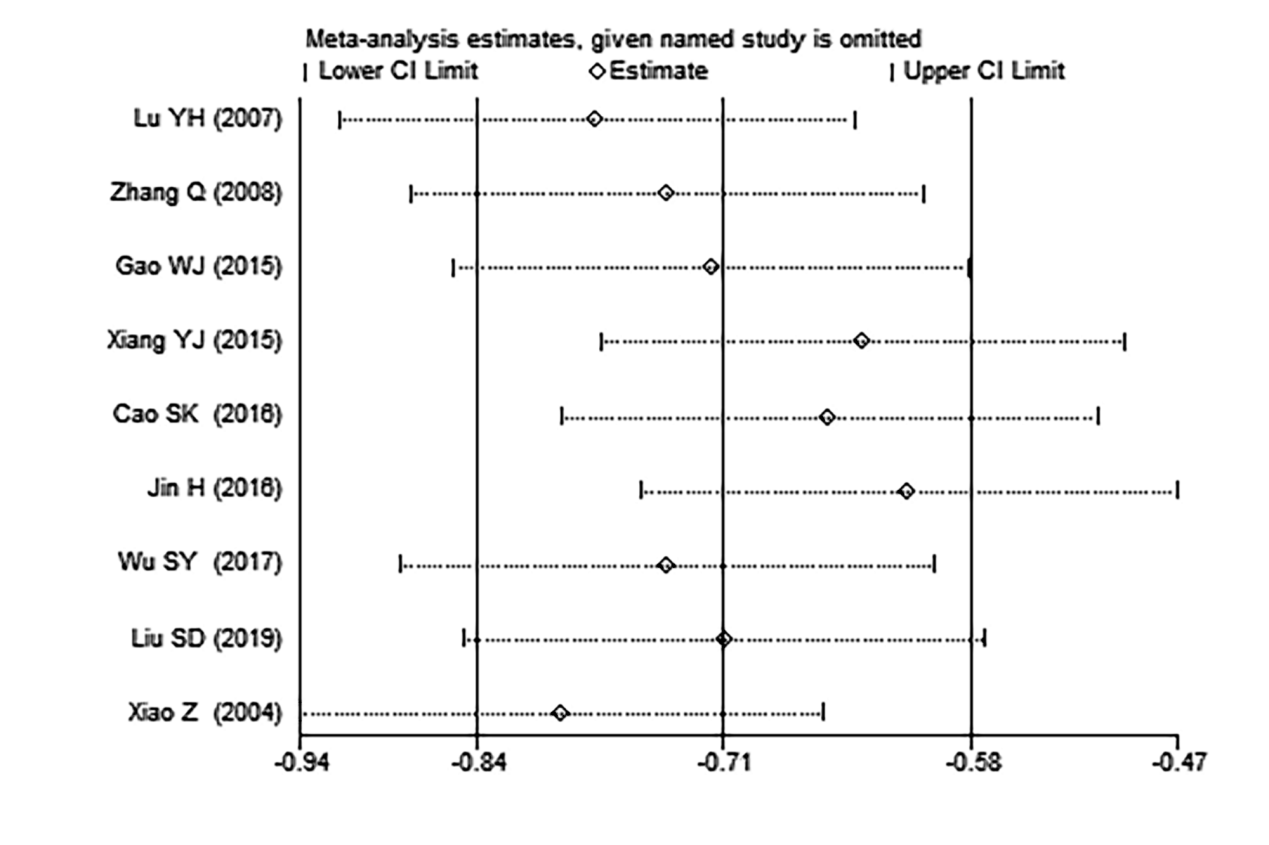
**

# Supplemental Digital Content (Figure S10): Sensitivity analysis for SBP.

**
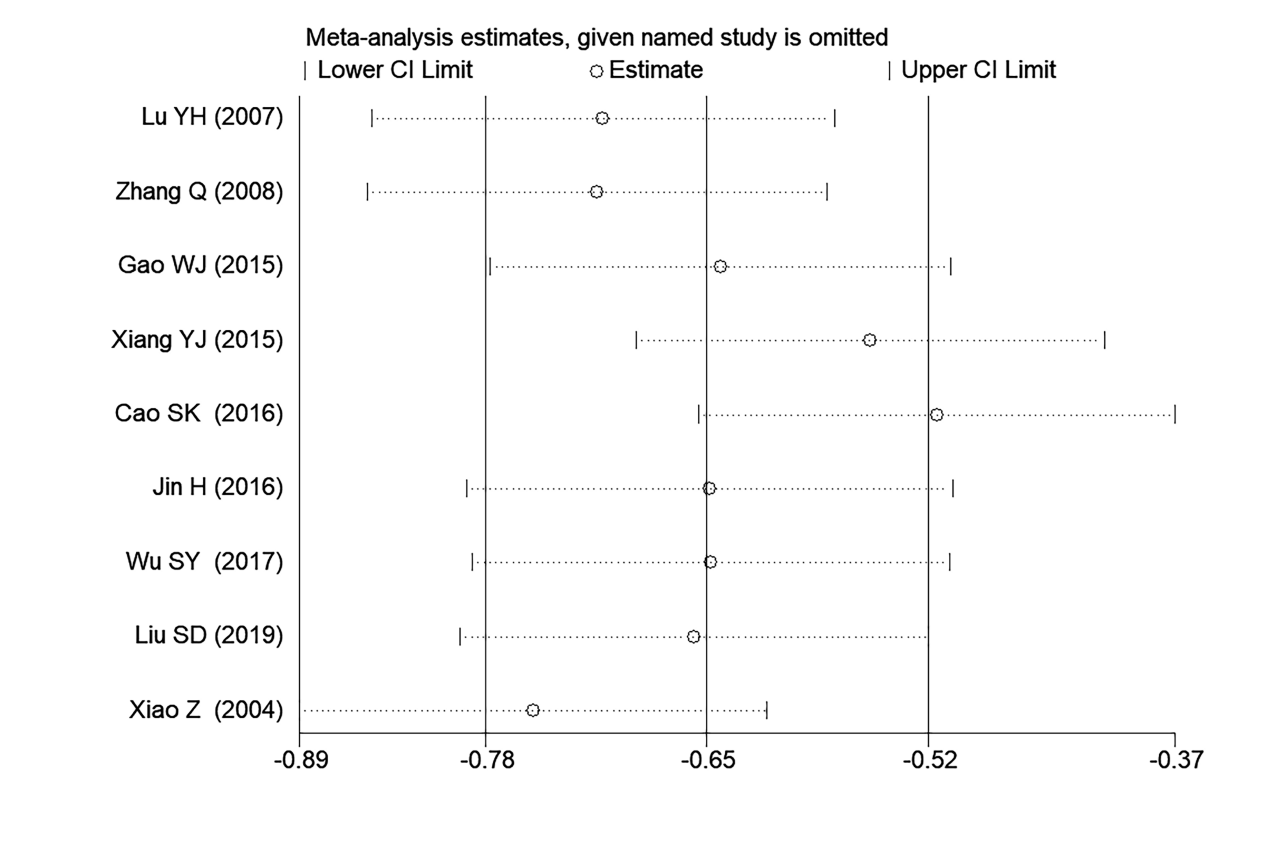
**

# Supplemental Digital Content (Figure S11): sensitivity analysis for DBP.

**
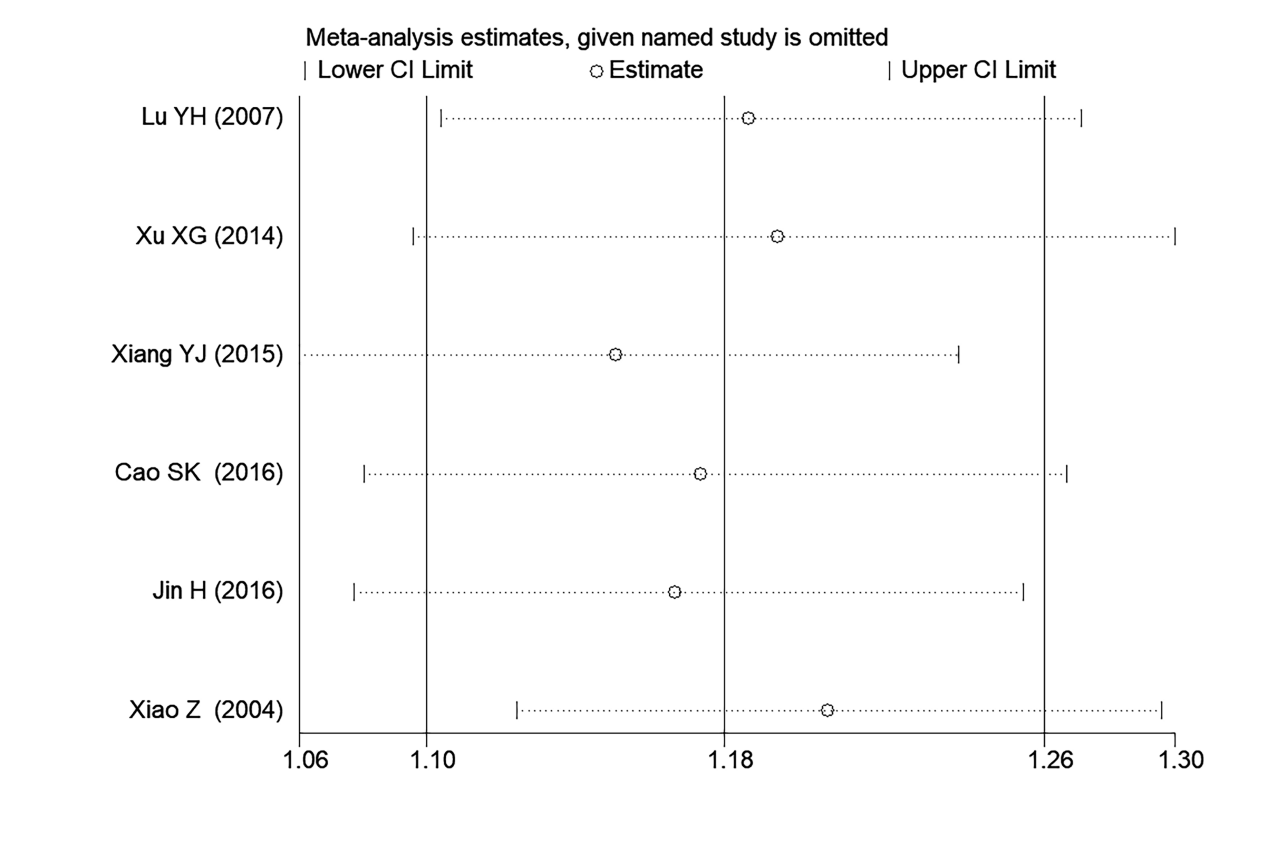
**

# Supplemental Digital Content (Figure S12): Sensitivity analysis for clinical efficacy.

**
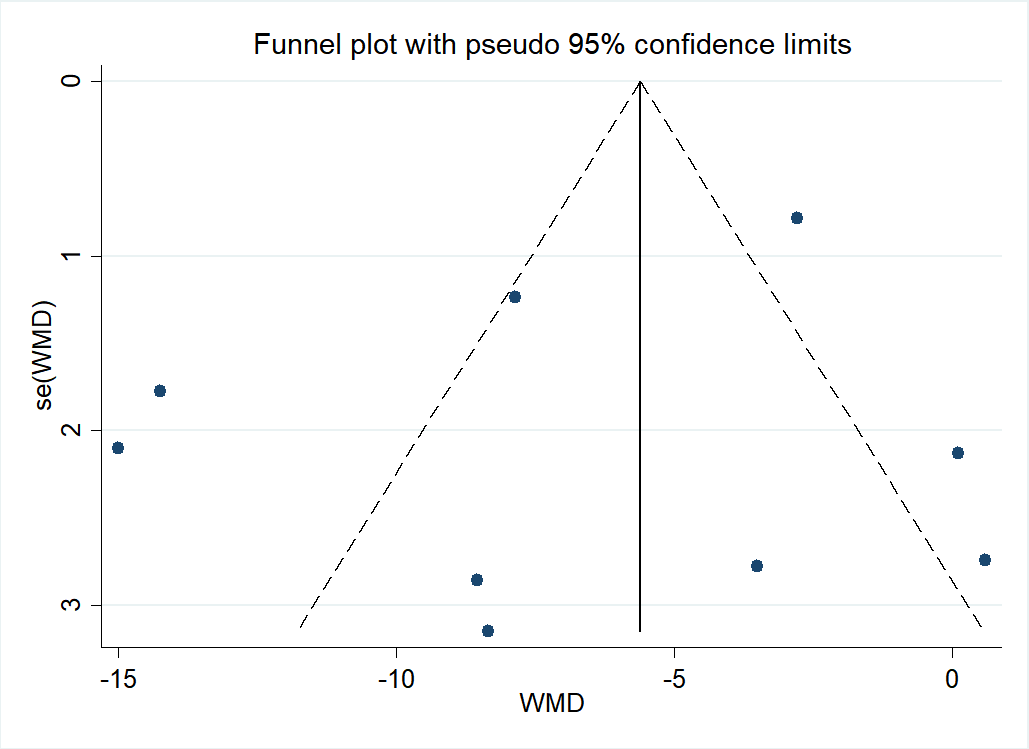
**

# Supplemental Digital Content (Figure S13): Funnel plot for SBP.

**
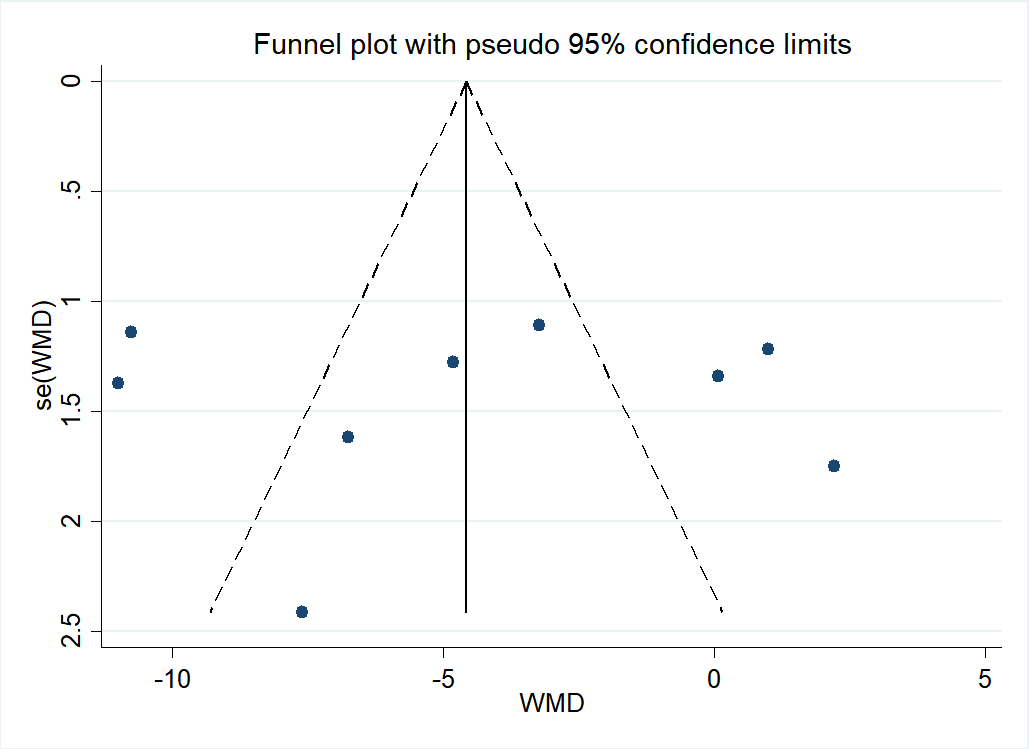
**

# Supplemental Digital Content (Figure S14): Funnel plot for DBP.

**
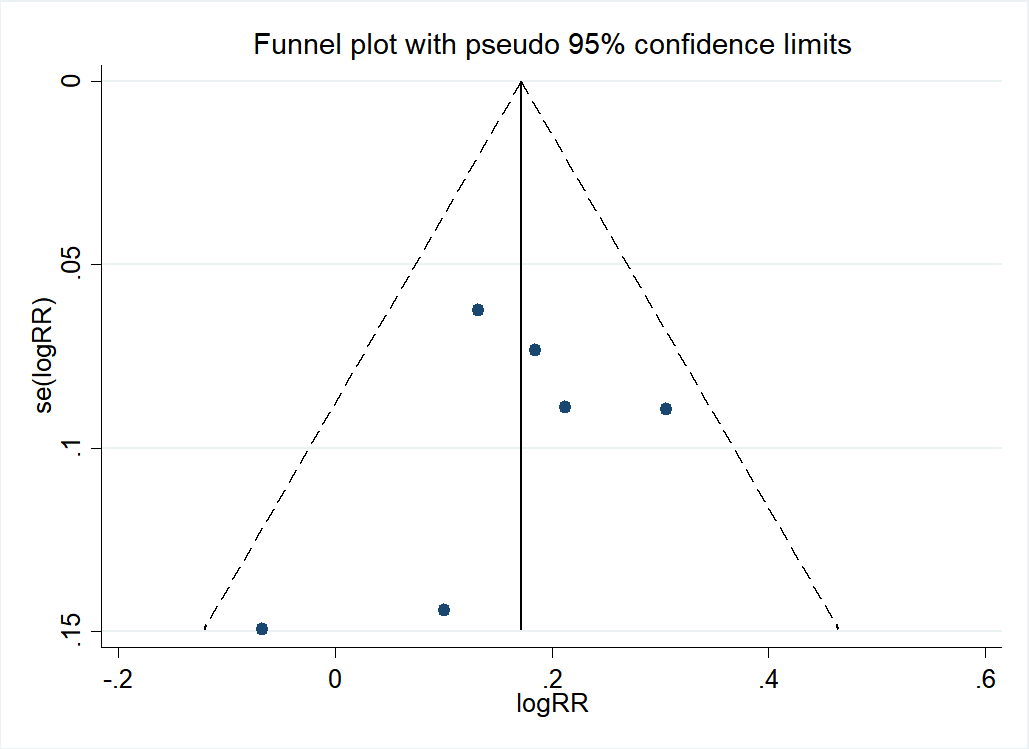
**

# Supplemental Digital Content (Figure S15): Funnel plot for clinical efficacy.
